# Failing to Forget: Inhibitory-Control Deficits Compromise Memory Suppression in Posttraumatic Stress Disorder

**DOI:** 10.1177/0956797615569889

**Published:** 2015-05

**Authors:** Ana Catarino, Charlotte S. Küpper, Aliza Werner-Seidler, Tim Dalgleish, Michael C. Anderson

**Affiliations:** 1 Medical Research Council, Cognition and Brain Sciences Unit, Cambridge, United Kingdom; 2 Department of Clinical Psychology and Psychotherapy, Freie Universität Berlin; 3 Cambridgeshire and Peterborough NHS Foundation Trust (CPFT), Cambridge, United Kingdom; 4 Behavioural and Clinical Neurosciences Institute, University of Cambridge

**Keywords:** memory suppression, forgetting, trauma, PTSD, inhibitory control, thought control

## Abstract

Most people have experienced distressing events that they would rather forget. Although memories of such events become less intrusive with time for the majority of people, those with posttraumatic stress disorder (PTSD) are afflicted by vivid, recurrent memories of their trauma. Often triggered by reminders in the daily environment, these memories can cause severe distress and impairment. We propose that difficulties with intrusive memories in PTSD arise in part from a deficit in engaging inhibitory control to suppress episodic retrieval. We tested this hypothesis by adapting the think/no-think paradigm to investigate voluntary memory suppression of aversive scenes cued by naturalistic reminders. Retrieval suppression was compromised significantly in PTSD patients, compared with trauma-exposed control participants. Furthermore, patients with the largest deficits in suppression-induced forgetting were also those with the most severe PTSD symptoms. These results raise the possibility that prefrontal mechanisms supporting inhibitory control over memory are impaired in PTSD.

Most people experience traumatic events that they would rather forget. Frequently, such forgetting is made difficult because stimuli in the environment resemble something from the trauma, eliciting intrusive remindings of what happened—an object in a drawer can remind people of someone lost because of death or a broken relationship; driving past a familiar road can remind people of a horrible accident they witnessed. For most trauma survivors, intrusions decline naturally over the first few months after trauma (Ehlers, 2010). However, for a small group of survivors, intrusive memories persist over extended periods in the form of both thoughts and images, causing marked functional impairment (Sherin & Nemeroff, 2011). This is a key feature of posttraumatic stress disorder (PTSD), a debilitating psychiatric condition that results from exposure to a severe traumatic event and is characterized by persisting clinical symptoms such as intrusive memories, flashbacks, avoidance, and emotional numbing (American Psychiatric Association, 2013).

Avoidance strategies are commonly employed by people with PTSD to evade reminders of trauma and mitigate the distress that consequent intrusions cause. However, although avoidance of the reminders themselves is one means to reduce memory intrusions, previous research has shown that people are often able to voluntarily suppress unwanted memories even when confronted with a reminder, a phenomenon known as suppression-induced forgetting (Anderson & Green, 2001; Anderson & Hanslmayr, 2014; Anderson et al., 2004; Benoit & Anderson, 2012; Depue, Banich, & Curran, 2006; Depue, Curran, & Banich, 2007; Gagnepain, Henson, & Anderson, 2014; Hertel & Mahan, 2008; Levy & Anderson, 2012). Large individual differences in this suppression ability have been observed, and these findings suggest that relative difficulties with suppression may underlie memory-control difficulties in conditions like PTSD (Hertel & Gerstle, 2003; Levy & Anderson, 2008; Marzi, Regina, & Righi, 2014). Do difficulties with intrusive memories in PTSD arise in part from compromised retrieval suppression? To address this question, in the present study (the first of its kind, to our knowledge), we examined suppression-induced forgetting in individuals with PTSD.

We wanted to look at people’s ability to suppress memories of aversive scenes in a naturalistic way. So, rather than using stimuli such as neutral word pairs or arbitrary face-scene associations (Depue et al., 2006; Depue et al., 2007; van Schie, Geraerts, & Anderson, 2013), we asked participants to study object-scene pairs. The cue objects resembled objects embedded in the aversive target scenes, thus serving as powerful triggers to remembering the scenes themselves. The stimuli therefore provided a meaningful analogue to situations in which traumatic intrusions are triggered by environmental elements related to the trauma (Ehlers, 2010; Küpper, Benoit, Dalgleish, & Anderson, 2014). At the end of the experiment, we tested memory recall for all the scenes. A previous study using the same think/no-think (TNT) paradigm found that healthy volunteers could suppress memories of aversive scenes, but suppression ability was weaker in those with lower scores on a measure of self-perceived thought-control ability (Küpper et al., 2014). This study provides a robust platform for examining memory suppression in PTSD.

We aimed to use this novel paradigm to investigate how well trauma-exposed individuals, with and without PTSD, could suppress retrieval of aversive images when triggered by powerful reminders. We propose that the ability to engage inhibitory control to support retrieval suppression is compromised in people with PTSD and that this deficit poses a central problem in regulating intrusive memories. Our hypothesis receives support from evidence for inhibitory-control deficits in PTSD as measured by motor response-inhibition tasks such as the go/no-go and stop-signal tasks (Falconer et al., 2008), as well as by other tasks that putatively involve memory inhibition, such as directed-forgetting and retrieval-induced-forgetting tasks (Amir, Badour, & Freese, 2009; McNally, 1998; McNally, Metzger, Lasko, Clancy, & Pitman, 1998). However, although the latter findings are highly promising as evidence for deficient memory control, their relevance to controlling intrusive memories is arguably indirect: Retrieval-induced forgetting concerns the tendency for retrieval of some items to incidentally inhibit other competing memories; directed forgetting concerns the ability to forget an immediately preceding event, in some cases by terminating encoding. Though both tasks may involve inhibitory control, neither addresses the situation most clearly relevant to combating intrusive memories of trauma: confronting unwelcome reminders and needing to suppress episodic retrieval to prevent awareness of an intrusive memory. Studying retrieval suppression of aversive images may therefore be particularly relevant to understanding key symptoms of PTSD, in which the high prevalence of intrusive memories and images results in significant distress and functional impairment.

We hypothesized that individuals with PTSD, compared with trauma-exposed individuals who have never developed PTSD, are less able to suppress retrieval of aversive scenes when confronted with powerful reminders. Additionally, given previous findings of a correlation between suppression-induced forgetting and self-perceived thought-control ability (Küpper et al., 2014), we predicted that lower retrieval-suppression abilities are associated with more severe PTSD symptoms and also with lower self-perceived thought-control abilities. These findings might help answer the key question of why some individuals recover naturally after trauma, whereas others continue to experience distressing intrusions that contribute to the development and persistence of PTSD.

## Method

### Participants

Eighteen individuals with a current diagnosis of PTSD (11 females; mean age = 34 years, *SD* = 13) and a control group of 18 trauma-exposed individuals with no current or past history of PTSD (11 females; mean age = 37 years, *SD* = 14) were recruited from the local community and departmental participant panels through print advertisements. Sample size was calculated with an a priori power analysis, using the effect sizes reported by Küpper et al. (2014), who used identical procedures, materials, and dependent measures. We determined that a minimum sample size of 7 per group would be necessary for 95% power to detect an effect. Additionally, our choice of a sample size of 18 per group is consistent with sample sizes used in previous think/no-think (TNT) studies (Anderson et al., 2004; Benoit & Anderson, 2012; Benoit, Hulbert, Huddleston, & Anderson, 2015; Gagnepain et al., 2014; Küpper et al., 2014). Diagnostic status was determined using the Structured Clinical Interview for the DSM-IV Axis I Disorders (First, Spitzer, Gibbon, & Williams, 1996). Exclusion criteria for PTSD participants included a current diagnosis of psychosis or borderline personality disorder. Control participants with any current psychiatric disorder were also excluded.

The range of participants’ trauma experiences was similar between the two groups and included experiencing or witnessing serious accidents (PTSD: *n* = 4; control: *n* = 9), violence (PTSD: *n* = 2; control: *n* = 3), sexual assault (PTSD: *n* = 2; control: *n* = 1), life-threatening illnesses (PTSD: *n* = 3; control: *n* = 4), combat situations (PTSD: *n* = 1), and traumatic death of family members or close friends (PTSD: *n* = 6; control: *n* = 1). Time since trauma was matched across the groups and varied from 3 months to more than 5 years. We recruited a heterogeneous trauma group because our hypothesis was that a key symptom of the disorder—intrusive, difficult-to-control memories—reflects at least a partial deficit in retrieval suppression, which ought to transcend trauma type.

All participants were native English speakers with no history of severe head injury, neurological disease, or learning disability. Further demographic details are provided in Table 1. This study received ethical approval from the Cambridgeshire and Peterborough Research Ethics Committee. All participants provided written informed consent.

**Table 1.** Comparison of the Demographic and Clinical Characteristics of the Posttraumatic Stress Disorder (PTSD) and Control Groups

Characteristic	PTSD group (*n* = 18)	Control group (*n* = 18)	Group comparison
Age (mean in years)	34.2 (13.4)	36.8 (14.2)	*t*(34) = −0.57, *p* = .57, *d* = −0.19, 95% CI = [–11.94, 6.72]
Gender	11 females, 7 males	11 females, 7 males	
PDS (mean score)	29.4 (7.4)	5.9 (5.4)	*t*(34) = 10.84, *p* < .001, *d* = 3.63, 95% CI = [19.09, 27.91]
IES-R (mean score)	44.2 (17.2)	11.0 (13.4)	*t*(34) = 6.48, *p* < .001, *d* = 2.15, 95% CI = [22.80, 43.64]
Intrusion	15.6 (7.9)	4.2 (5.1)	*t*(29.073) = 5.12, *p* < .001, *d* = 1.71, 95% CI = [6.81, 15.86]
Avoidance	15.7 (7.7)	5.2 (7.5)	*t*(34) = 4.11, *p* < .001, *d* = 1.38, 95% CI = [5.28, 15.61]
Hyperarousal	13.0 (5.4)	1.6 (3.4)	*t*(34) = 7.60, *p* < .001, *d* = 2.53, 95% CI = [8.38, 14.51]
BDI-II (mean score)	25.4 (13.8)	7.2 (5.5)	*t*(22.212) = 5.20, *p* < .001, *d* = 1.73, 95% CI = [10.93, 25.41]
STAI-T (mean score)	54.1 (9.0)	39.3 (8.3)	*t*(34) = 5.04, *p* < .001, *d* = 1.71, 95% CI = [7.43, 17.46]
STAI-S (mean score)	40.1 (7.4)	31.2 (6.4)	*t*(33) = 3.81, *p* = .001, *d* = 1.29, 95% CI = [4.15, 13.64]
TCAQ (mean score)	55.7 (14.7)	83.9 (14.6)	*t*(34) = −5.79, *p* < .001, *d* = −1.92, 95% CI = [–38.14, –18.31]
NART (mean score)	31.3 (7.5)	34.2 (6.0)	*t*(34) = −1.31, *p* = .20, *d* = −0.43, 95% CI = [–7.53, 1.64]

Note: Standard deviations are given in parentheses. CI = confidence interval for the group difference; PDS = Posttraumatic Stress Diagnostic Scale (Foa, 1995); IES-R = Impact of Event Scale–Revised (Weiss, 2007); BDI-II = Beck Depression Inventory-II (Beck, Steer, & Brown, 1996); STAI-T = Trait score on the State-Trait Anxiety Inventory (Spielberger, Gorsuch, Lushene, Vagg, & Jacobs, 1983); STAI-S = State score on the State-Trait Anxiety Inventory (Spielberger et al., 1982); TCAQ = Thought Control Ability Questionnaire (Luciano, Algarabel, Tomás, & Martínez, 2005); NART = National Adult Reading Test (Nelson, 1982).

### Materials

The TNT memory task involved asking participants initially to study various object-scene pairs. Next, in the main phase of the experiment, the cue objects were presented, and participants were asked to either recall or suppress their memories for the associated scenes. At the final test, participants were shown the cue objects and asked to provide brief descriptions of the associated scenes.

**Fig. 1.**
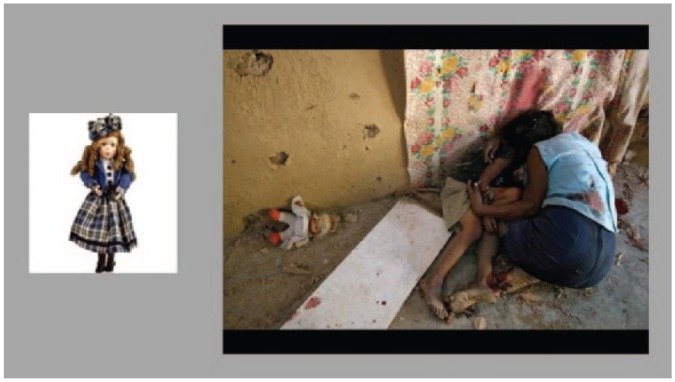
A representative object-scene pair consisting of a neutral cue object and an unpleasant scene. Each cue object was chosen to resemble an item that was naturally embedded as an incidental detail in the associated scene.

The stimuli used were 60 object-scene pairs: 48 critical pairs and 12 fillers (Fig. 1). The scenes for these pairs were emotionally negative images taken from the International Affective Picture System (Lang, Bradley, & Cuthbert, 2008) and online sources. We used negative emotional material rather than trauma-specific scenes because our hypothesis was that there is a generic inability to inhibit memories for aversive information in PTSD. The cue objects were colored photographs of familiar, neutral objects (taken from Brady, Konkle, Alvarez, & Oliva, 2008). Each cue object was chosen to resemble an item that was already naturally embedded as an incidental detail in its paired scene but not intrinsically related to the gist of the scene. This prevented guessing of the scenes during later recall. The 48 critical pairs were divided into three sets (referred to as sets A, B, and C) that were matched on salience of the cues, as well as the emotional valence and arousing nature of the scenes (Küpper et al., 2014). On a scale from 1 (*not salient*) to 5 (*very salient*), the mean rated salience of the cues was 2.7 for set A, 2.9 for set B, and 2.4 for set C; on a scale from 1 (*unpleasant*) to 9 (*pleasant*), the mean rated emotional valence of the scenes was 3.5 for set A, 3.3 for set B, and 3.3 for set C; and on a scale from 1 (*unarousing*) to 9 (*arousing*), the mean arousal rating of the scenes was 4.8 for set A, 4.7 for set B, and 4.8 for set C. Assignment of the sets to the three conditions (see the TNT Procedure section) was counterbalanced across participants.

At the end of the experiment, participants filled out the following questionnaires. The Thought Control Ability Questionnaire (TCAQ; Luciano, Algarabel, Tomás, & Martínez, 2005) is a 25-item questionnaire that assesses the self-perceived ability to exert control over thought intrusions; higher scores reflect better control ability. The Beck Depression Inventory-II (BDI-II; Beck, Steer, & Brown, 1996) is a self-administered questionnaire measuring the intensity of depressive symptoms. The Impact of Event Scale–Revised (IES-R; Weiss, 2007) is a self-report measure assessing the magnitude of symptomatic response, in the past 7 days, to a specific traumatic life event—in this case, the participant’s most significant trauma. The State-Trait Anxiety Inventory (STAI; Spielberger, Gorsuch, Lushene, Vagg, & Jacobs, 1983) is a self-administered questionnaire assessing trait and state anxiety. The Posttraumatic Stress Diagnostic Scale (PDS; Foa, 1995) is a self-administered questionnaire designed to aid in the detection and diagnosis of trauma-related symptoms. The National Adult Reading Test (NART; Nelson, 1982) is a researcher-administered test used to estimate an individual’s level of intellectual ability.

### TNT procedure

**Fig. 2.**
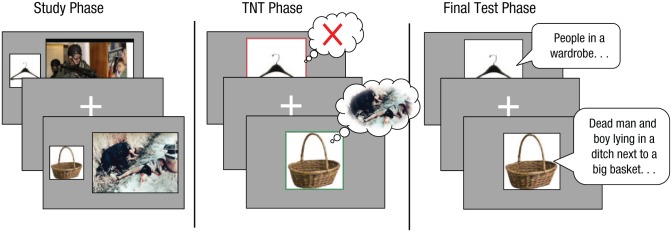
Schematic representation of the experimental procedure. In the study phase, participants encoded cue-target pairs. During the think/no-think (TNT) phase, participants were instructed to directly suppress memories associated with cue objects presented inside a red frame and to recall memories associated with cue objects presented inside a green frame. Finally, in the test phase, participants were asked to remember and verbally describe all the scenes that they had previously recalled (recall items) or suppressed (suppress items), as well as all the scenes that they had initially learned but had not seen during the TNT phase (baseline items).

We adapted the TNT procedure, developed by Anderson and Green (2001), to study the suppression of aversive scenes. The paradigm had three phases: study phase, TNT phase, and final test phase (Fig. 2).

#### Study phase

Participants started by studying all 60 object-scene pairs, which were presented for 6 s each in a blocked randomized order. Test-feedback cycles then followed. On each test trial, participants were shown a cue object and indicated, by pressing the “yes” or “no” button, whether they could recall the scene with which it had been paired. If they answered “yes,” three scenes were presented, and they were asked to select the correct one. Participants then were shown the correct object-scene pair again for 2.5 s, as feedback to enhance encoding. The testing cycled through all items repeatedly until participants reached a set criterion of at least 60% correct recognition (all succeeded within four cycles). When they reached this criterion, a final test cycle (without feedback) including all pairs assessed which pairs had been learned.

#### TNT phase

In this phase, participants were shown the cue objects alone. Each object appeared for 3 s in the center of the screen, surrounded by a colored frame, and was followed by a fixation cross of varying duration (interstimulus interval = 2 s ± 600 ms). Participants were asked to suppress the associated scene when an object was surrounded by a red frame (no-think trial) and to recall the associated scene when an object was surrounded by a green frame (think trial). At the start of this phase, participants were given direct suppression instructions for no-think trials (Benoit & Anderson, 2012), which requested them to suppress the associated scene and additionally avoid any distracting thoughts from coming into awareness. These instructions reduce the use of thought substitution and self-distraction, which results in a better measure of the role of inhibitory control in memory suppression (Benoit & Anderson, 2012). For recall trials, participants were instructed to recall the scene in as much detail as possible. Practice trials using filler items were presented to ensure that subjects understood the instructions. Following practice, participants were presented with 32 critical experimental cue objects: 16 in the recall condition and 16 in the suppress condition. The critical trials were split into five blocks, and each of these 32 objects was presented twice in each block. Thus, each associated scene was suppressed or recalled 10 times over the course of these trials. The remaining 16 critical object-scene pairs were baseline items; that is, they were learned in the study phase but were not presented in the TNT phase.

#### Final test phase

Participants’ memory for all critical scenes was tested after the TNT phase. All cue objects (from the recall, suppress, and baseline conditions) were presented, one at a time and without a colored frame. On each trial, participants were given 15 s to verbally describe the associated scene in as much detail as possible, so that it could be uniquely identified. The descriptions were recorded for later transcription.

### Dependent measures

Participants’ descriptions were scored on three dependent measures assessing quantitative and qualitative aspects of the memories. For the identification measure, a description was scored as correct if it included enough detail that the specific scene could be uniquely identified (Depue et al., 2006). For the details measure, each description was divided into small, meaningful segments conveying independent information, and the number of correct details was counted. Finally, gist was defined as any element pertaining to the central story of a scene that could not be changed or excluded without changing the main theme. Prior to the experiment, two independent judges determined two to four specific elements that contained the general gist of each scene. Descriptions were scored as correct on the gist measure if they included all necessary elements of the scene.

All descriptions were scored by two independent raters who were blind to the conditions. Interrater agreement was high—identification: *r* = .99; details: *r* = .95; gist: *r* = .93.

### Data analysis

Suppression-induced forgetting (lower memory performance for suppress compared with baseline items) was assessed using a mixed analysis of variance (ANOVA) with condition (baseline, suppress) as a within-subjects factor and group (PTSD, control) and set assignment (i.e., which of sets A, B, and C was assigned to each condition) as between-subject factors. Facilitation effects (better memory for recall compared with baseline items) were assessed using the same model, but the levels for condition were instead baseline and recall. ANOVAs were performed separately for each dependent measure (identification, gist, and details). Effect sizes (η_*p*_^2^ or Cohen’s *d*) and 95% confidence intervals (CIs) are reported. Nonsignificant effects were further explored through Bayesian analysis, by computing whether Bayes factors favored the alternative or null hypothesis.

We report correlations between the dependent variable showing the strongest suppression-induced-forgetting effect in the primary analysis (the details measure) and TCAQ and PDS scores (though correlations with all memory measures were similar). Self-perceived thought-control ability as measured by the TCAQ and PTSD symptomatology as measured by the PDS were chosen as main predictors of interest for the correlation analysis to test our hypothesis that suppression-induced forgetting is related to thought control in daily life, as well as to the severity of PTSD symptoms. We also calculated semipartial correlations between our details measure and TCAQ and PDS scores covarying out BDI-II scores in order to explore the role of depression symptoms as a possible confounding factor, given their high prevalence of comorbidity with PTSD. Rank correlations were used when data differed significantly from normality. Alpha was set at .05.

All statistical analyses were repeated using final-test data conditionalized on correct initial learning of the object-scene pairs. A pair was judged as initially learned if it was recalled correctly in the final test cycle of the study phase.

## Results

### Description of the samples

Demographic and clinical information about the participants is presented in Table 1. As expected, the two groups differed significantly in their scores for clinical measures tapping into PTSD symptomatology and symptoms that commonly co-occur with PTSD, such as depression and anxiety.

### TNT-task performance

**Fig. 3.**
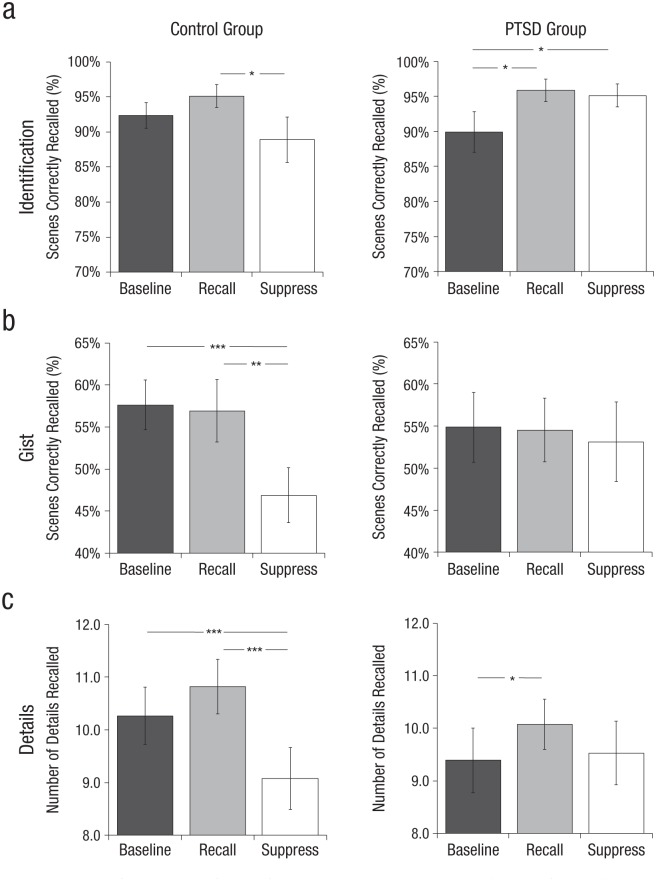
Memory performance in the final test for the trauma-exposed control group (*n* = 18; left column) and the posttraumatic stress disorder (PTSD) group (*n* = 18; right column). Results are shown separately for baseline, recall, and suppress items for each dependent measure: (a) identification, (b) gist, and (c) details. Suppression-induced forgetting is when significantly fewer suppress than baseline items are remembered. Memory facilitation is when significantly more recall than baseline items are remembered. Error bars represent ±1 *SE*. Asterisks indicate significant differences between conditions (*p < .05; **p < .01; ***p < .001).

If, as we hypothesized, inhibitory control over memory retrieval is impaired in PTSD, control participants should exhibit larger suppression-induced forgetting than PTSD patients do. This predicted pattern was confirmed for all three dependent measures (Fig. 3).

#### Identification

A significant group difference was found in suppression-induced forgetting, as revealed by a significant condition-by-group interaction, *F*(1, 30) = 7.556, *p* = .010, η_*p*_^2^ = .201. This interaction was driven by a significant inversion of suppression-induced forgetting in the PTSD group (i.e., better memory performance for suppress items compared with baseline items; *M* = −5.2%), *F*(1, 15) = 5.288, *p* = .036, η_*p*_^2^ = .261 (Bayes factor favoring the alternative hypothesis = 2.09), in the absence of significant suppression in the control group (*M* = 3.5%, n.s.). Although the PTSD group showed a significant facilitation effect (*M* = 5.9%), *F*(1, 15) = 7.525, *p* = .015, η_*p*_^2^ = .334, and the control group did not (*M* = 2.8%, n.s.), there was no significant group difference in facilitation, *F*(1, 30) = 1.215, *p* = .279, η_*p*_^2^ = .039 (Fig. 3a). The absence of reliable facilitation and suppression effects in the control group may have been due to ceiling effects.

#### Gist

A significant group difference in suppression-induced forgetting was observed, as indicated by a significant condition-by-group interaction, *F*(1, 30) = 4.573, *p* = .041, η_*p*_^2^ = .132. This interaction was driven by significant suppression-induced forgetting in the control group (*M* = 10.8%), *F*(1, 15) = 22.123, *p* < .001, η_*p*_^2^ = .596, in the absence of significant suppression-induced forgetting in the PTSD group (*M* = 1.7%), *F*(1, 15) = 0.297, *p* = .594, η_*p*_^2^ = .019. A Bayesian analysis indicated strong evidence in favor of the null hypothesis, confirming the absence of suppression-induced forgetting in the PTSD group (Bayes factor in favor of the null hypothesis = 3.74). No significant facilitation effect was found in either group (PTSD: *M* = −0.3%; control: *M* = −0.7%; Fig. 3b).

#### Details

As for the two previous measures, a significant group difference in suppression-induced forgetting was observed, established by a significant condition-by-group interaction, *F*(1, 30) = 14.231, *p* = .001, η_*p*_^2^ = .322. This interaction was driven once again by a significant suppression effect in the control group (*M* = 1.19), *F*(1, 15) = 33.548, *p* < .001, η_*p*_^2^ = .691, in the absence of significant suppression in the PTSD group (*M* = −0.14), *F*(1, 15) = 0.224, *p* = .643, η_*p*_^2^ = .015. Again, a Bayesian analysis indicated strong evidence in favor of the null hypothesis, confirming the absence of suppression-induced forgetting in the PTSD group (Bayes factor in favor of the null hypothesis = 3.70). Although the PTSD group showed a significant facilitation effect (*M* = 0.68), *F*(1, 15) = 5.575, *p* = .032, η_*p*_^2^ = .271, and the control group showed only a marginally significant effect (*M* = 0.56), *F*(1, 15) = 4.368, *p* = .054, η_*p*_^2^ = .226, the difference between the groups was not significant, *F*(1, 30) = 0.100, *p* = .754, η_*p*_^2^ = .003 (Fig. 3c).

#### Measures of overall learning

No significant group differences were found in initial learning of the pairs (control: *M* = 87%, *SD* = 11%; PTSD: *M* = 93%, *SD* = 7%). The groups also did not differ in accuracy for baseline items, whether assessed by the identification measure (control: *M* = 92%, *SD* = 8%; PTSD: *M* = 90%, *SD* = 12%), the gist measure (control: *M* = 58%, *SD* = 13%; PTSD: *M* = 55%, *SD* = 18%), or the details measure (control: *M* = 10.27, *SD* = 2.38; PTSD: *M* = 9.39, *SD* = 2.62). Finally, the groups also did not differ in accuracy for recall items, whether assessed by the identification measure (control: *M* = 95%, *SD* = 7%; PTSD: *M* = 96%, *SD* = 7%), the gist measure (control: *M* = 57%, *SD* = 16%; PTSD: *M* = 55%, *SD* = 16%), or the details measure (control: *M* = 10.82, *SD* = 2.26; PTSD: *M* = 10.07, *SD* = 2.03). These results show that the two groups had comparable learning and baseline memory performance, and that there was no clear bias toward better recall of the emotionally negative scenes in the PTSD group.

### Thought Control Ability Questionnaire

**Fig. 4.**
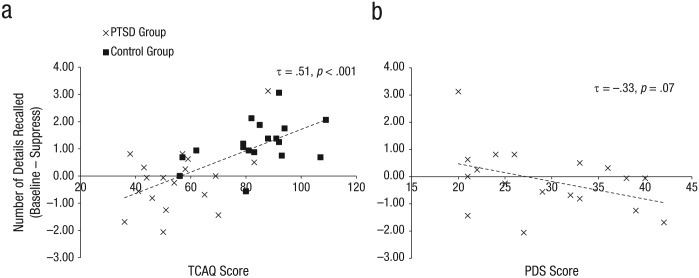
Scatter plots illustrating the relation between suppression-induced forgetting as assessed by the details measure (number of details for baseline items – number of details for suppress items) and (a) self-perceived thought-control ability across the entire sample and (b) severity of posttraumatic stress disorder (PTSD) symptoms in the PTSD group. Thought-control ability was assessed by the Thought Control Ability Questionnaire (TCAQ; Luciano, Algarabel, Tomás, & Martínez, 2005), and PTSD symptoms were assessed by the Posttraumatic Stress Diagnostic Scale (PDS; Foa, 1995).

The control group reported higher self-perceived thought-control ability than did the PTSD group (Table 1), although TCAQ scores across the whole sample showed a continuous normal distribution. Across all participants, suppression-induced forgetting on our details measure and TCAQ scores had a robust positive correlation (Kendall’s τ = .5, *p* < .001; Fig. 4a). This correlation remained significant when we covaried out BDI-II scores using semipartial correlation analyses (τ = .34, *p* = .004), which suggests that the correlation was not simply a function of participants’ current symptom levels.

### Posttraumatic Stress Diagnostic Scale

The PTSD group reported more severe PTSD symptoms than did the control group (Table 1). As expected, the distribution of PDS scores across groups was not continuous, given that the control group was mostly at floor for this measure. For this reason, correlation analyses were performed only within the PTSD group. A marginally significant negative correlation was found between suppression-induced forgetting on the details measure and PDS scores in the PTSD group (Kendall’s τ = −.33, *p* = .07; Fig. 4b). This correlation was rendered significant when we covaried out BDI-II scores using semipartial correlation analyses (τ = −.35, *p* = .049).

### The effects of depressive symptoms

The PTSD group had significantly higher BDI-II scores than did the control group (see Table 1). Because depressive symptoms have been associated with deficits in suppression-induced forgetting (Hertel & Gerstle, 2003; Joormann, Hertel, LeMoult, & Gotlib, 2009), the suppression deficit in the PTSD group may reflect effects of this comorbidity. As just noted, however, the negative correlation between PTSD symptom severity and suppression-induced forgetting actually strengthened after BDI-II scores were partialed out, which suggests that depressive symptoms may not have been the main factor underlying the observed deficit.

To scrutinize this issue further, we performed a median split within each group according to BDI-II scores. We found that within the PTSD group, suppression-induced forgetting (i.e., difference in memory performance between baseline and suppress items) was never found in either the low-BDI-II group (mean BDI-II score = 15.1, *SD* = 8.8) or the high-BDI-II group (mean BDI-II score = 35.7, *SD* = 9.4), regardless of the dependent measure examined—identification: *M* = −5% for the low group and −6% for the high group; gist: *M* = 7% for the low group and −3% for the high group; details: *M* = −0.03 for the low group and −0.24 for the high group. For the control group, in contrast, suppression-induced forgetting was observed in both the low-BDI-II group (mean BDI-II score = 3.6, *SD* = 3.1) and the high-BDI-II group (mean BDI-II score = 10.9, *SD* = 4.9), despite the small sample sizes (*n* = 9). Specifically, both groups showed significant suppression-induced forgetting on the gist measure—low group: *M* = 13%, *F*(1, 6) = 30.388, *p* = .001, η_*p*_^2^ = .835; high group: *M* = 8%, *F*(1, 6) = 8.641, *p* = .026, η_*p*_^2^ = .590. They also showed highly significant suppression-induced forgetting on the details measure—low group: *M* = 1.35, *F*(1, 6) = 11.631, *p* = .014, η_*p*_^2^ = .660; high group: *M* = 1.03, *F*(1, 6) = 27.222, *p* = .002, η_*p*_^2^ = .819. Note, though, that the high-BDI-II group showed numerically less suppression-induced forgetting than did the low-BDI-II group.

When we more closely matched depression symptoms by comparing the low-BDI-II PTSD group with the high-BDI-II control group, we observed a marginally significant group-by-condition interaction for the details measure, *F*(1, 16) = 4.032, *p* = .062, η_*p*_^2^ = .201. Significant suppression-induced forgetting was observed for the high-BDI-II control group, but not for the low-BDI-II PTSD group, despite BDI-II scores that did not differ appreciably. Taken together, these results suggest that the observed deficits in suppression-induced forgetting in the PTSD group are unlikely to solely reflect the effects of depressive symptomatology on memory control.

### Conditionalized final-test data

Analyses of the data conditional on correct initial learning yielded similar results, except in the case of the identification measure. In that analysis, the group difference in suppression-induced forgetting only approached significance, *F*(1, 30) = 3.869, *p* = .058, η_*p*_^2^ = .114.

## Discussion

Inhibitory control mechanisms serve an important role in various domains of cognition. If faced with a falling object, one’s first instinct is to try and catch it. However, if the object is a sharp knife, one suppresses this prepotent motor response, letting the knife fall on the floor to preserve one’s physical well-being. A similar inhibitory response occurs with memory. When people are confronted with reminders of unpleasant experiences, memories flood awareness, and attempts to stop the retrieval process follow quickly. Previous research suggests that suppressing retrieval of intrusive memories reduces their accessibility (Anderson & Green, 2001; Küpper et al., 2014), raising the possibility that, just as inhibiting actions helps preserve physical well-being, inhibiting memories may preserve emotional well-being.

We hypothesized that deficient inhibitory control in people with PTSD compromises their ability to suppress episodic retrieval, causing persistent difficulties with intrusive memories. Supporting this hypothesis, our results showed that for all measures, suppression-induced forgetting was diminished in the PTSD group compared with the control group. We also observed a negative correlation between suppression-induced forgetting and PTSD symptom severity, which indicates that retrieval suppression is most compromised in people with the most severe symptoms. This finding is consistent with clinical observations of more frequent intrusions in cases of more severe PTSD (Ehlers, 2010; Sherin & Nemeroff, 2011). Finally, we found a large positive correlation between suppression-induced forgetting and self-reported thought-control ability, which suggests that this forgetting reflects mechanisms contributing to memory control in daily life (Küpper et al., 2014; Williams et al., 2010). Taken together, our results suggest that difficulties in suppressing memories of aversive scenes may be related to PTSD patients’ broader difficulties controlling intrusive memories outside the laboratory.

It is important to note that our two groups performed comparably during initial learning and also on the final test for baseline and recall items. Indeed, PTSD participants showed reliable facilitation for recall items. Thus, the group differences in suppression-induced forgetting cannot be explained by differences in overall performance or by a bias toward better memory for negative scenes in the PTSD group. Enhanced memory for think (recall) items was weak in both groups, however; this is a recurring observation in TNT studies using complex event and scene stimuli (Depue et al., 2007; Küpper et al., 2014; Stephens, Braid, & Hertel, 2013), and it remains to be understood. Clearly, however, the PTSD and control groups differed primarily in whether suppression impaired memory for suppress items, and our results are consistent with deficient inhibitory control over retrieval in people with PTSD. It would be useful for future studies to provide converging evidence that these suppression-induced forgetting effects reflect lingering inhibition of suppressed memories, as found in other work (Anderson & Green, 2001).

What remains unclear, however, is whether impaired memory control is caused by PTSD, or is a risk factor for its development. Although our data do not provide an answer to this question, our results show that trauma exposure, by itself, is not sufficient to impair memory suppression, as all participants had experienced trauma. It is noteworthy, however, that the correlation between self-perceived thought-control ability (TCAQ score) and suppression-induced forgetting remained significant after we covaried out a measure of emotional functioning (the BDI-II), which raises the possibility that deficient inhibitory control predisposes people to develop PTSD (Küpper et al., 2014). This possibility is consistent with evidence indicating that lower scores on cognitive-ability measures predict increased risk of developing PTSD (McNally & Shin, 1995), even in prospective designs (Breslau, Lucia, & Alvarado, 2006; Macklin et al., 1998). If deficient inhibitory control predisposes people to develop PTSD, TCAQ scores may provide a screening tool to identify those at greater risk.

Regardless of its origins, deficient retrieval suppression in PTSD may reflect disordered prefrontal control over memory-related brain areas. Retrieval suppression engages right dorsolateral prefrontal cortex (DLPFC) to reduce activity in the hippocampus (for a review, see Anderson & Hanslmayr, 2014; Anderson et al., 2004; Benoit & Anderson, 2012; Benoit et al., 2015; Depue et al., 2007; Gagnepain et al., 2014; Levy & Anderson, 2012; Paz-Alonso, Ghetti, Matlen, Anderson, & Bunge, 2009). Reports of structural and functional abnormalities in prefrontal cortex of people with PTSD (Fani et al., 2012; Lyoo et al., 2011; Moores et al., 2008; Shin et al., 2004; Yamasue et al., 2003), along with findings that they have deficient inhibitory control, suggest that they have difficulty engaging this DLPFC-hippocampal pathway, which causes the symptom of persistent reexperiencing. If these neural mechanisms are impaired in PTSD, then suppression may not only be ineffective but may actually increase symptom severity and persistence if intruding memories are enhanced, a finding confirmed by previous research (Brewin, 2011; Ehlers & Clark, 2000; Ehlers, Mayou, & Bryant, 1998; Mayou, Ehlers, & Bryant, 2002).

The present study shows that one can investigate suppression-induced forgetting in a laboratory environment in a way that is relevant to deficits experienced by people with PTSD. However, although we used a relatively naturalistic design, the aversive images elicited during the TNT task only approximate the intrusive memories people experience in real life. We can speculate that the latter may be either more or less vulnerable to suppression than the former. On the one hand, traumatic memories, usually associated with guilt, fear, or anger, may be more difficult to suppress than our aversive images. Additionally, PTSD symptoms such as negative self-appraisal may make suppressing intrusive memories difficult, increasing symptom severity (Meiser-Stedman, Dalgleish, Glucksman, Yule, & Smith, 2009). On the other hand, trauma survivors may be particularly motivated to suppress their intrusive memories, persisting in their suppression efforts over long periods, and thereby enhancing forgetting. Previous research indicates that cumulative suppression efforts totaling 1 min for a specific item (over a think/no-think task lasting 30 to 45 min) cause suppression-induced forgetting that can last from 24 hr to 1 week (Anderson & Huddleston, 2012). It is therefore possible that trauma survivors’ persisting efforts yield longer-lasting suppression-induced forgetting than our TNT task does.

The impact of long-term suppression efforts on symptom severity remains unexplored. It is widely believed that avoiding distressing memories exacerbates PTSD symptoms by keeping suppressed memories accessible. Cognitive behavioral therapy for PTSD is thought to be effective because it encourages patients to stop avoiding memories and to confront reminders until the traumatic memories become less distressing (Kar, 2011). We suggest an alternative possibility, proposing that there is an important distinction between avoiding reminders, on the one hand, and avoiding the memory (via suppression) given that a reminder is confronted, on the other. The former strategy should preserve memories by depriving people of opportunities to forget via inhibitory control, whereas avoidance by retrieval suppression is beneficial, if implemented effectively. Thus, like cognitive behavioral therapy, retrieval suppression forces people to confront reminders, but to learn to control awareness of their memories. One interesting hypothesis is that cognitive behavioral therapy is effective, in part, because confronting reminders and learning to redirect one’s thoughts relies on the inhibitory processes observed in the current experiment. If so, perhaps interventions may be devised to complement behavioral therapy by training PTSD patients to be more effective at memory control.

Another issue is our choice to use generically aversive materials rather than materials tailored to individual participants’ trauma experiences. This choice arose from our hypothesis that impaired retrieval suppression should not be limited to memories of the trauma itself, but should broadly affect memory control. Although our data support this hypothesis, a remaining question is whether retrieval-suppression deficits in PTSD extend to nonemotional material.

Another question for future research is whether deficient retrieval suppression extends to other psychiatric conditions. Although we focused on PTSD, retrieval-suppression deficits may reflect a transdiagnostic problem associated with other disorders in which ruminative tendencies are common and intrusive memories prevail, such as depression, generalized anxiety disorder, and obsessive-compulsive disorder (Hertel & Gerstle, 2003; Marzi et al., 2014; Patel et al., 2007; Speckens, Hackmann, Ehlers, & Cuthbert, 2007). Indeed, depressed participants show diminished suppression-induced forgetting (though not when given thought-substitution strategies; Joormann et al., 2009), as do ruminators regardless of depression symptomatology (Fawcett et al., 2015; Hertel & Gerstle, 2003). Whether this recurring pattern reflects a single transdiagnostic deficit with a common cause or a collection of disorder-specific deficits remains to be established. One must also consider whether worse suppression reflects a deficiency in memory control or, instead, a failure to sustain effort arising from a compromised emotional state. What is clear in the present study, however, is that deficient suppression occurred in PTSD patients regardless of their depression symptoms, and this suggests that depression is not a prerequisite for this deficit. This possibility should be confirmed in future work.

Although remembering past experiences serves an adaptive role, intrusive memories of negative events can severely affect emotional well-being. This is particularly true for people with PTSD, whose everyday functioning is severely impaired by recurrent intrusive memories of trauma. We used a naturalistic design to investigate, for the first time, voluntary retrieval suppression of aversive scenes in PTSD. Our findings suggest that the difficulties with intrusive memories in PTSD may be caused, in part, by deficient inhibitory control over retrieval. Moreover, by virtue of the correlation between deficits in inhibitory control and scores on clinical scales, our results affirm the relevance of retrieval suppression to memory-control deficits in PTSD and other disorders characterized by uncontrolled memories and thoughts. Along with the rapidly emerging literature on the neural mechanisms supporting retrieval suppression (for a review, see Anderson & Hanslmayr, 2014), the present findings open the door to a precise characterization of the neurobiological mechanisms underlying PTSD and other disorders of mnemonic control.

## Supplementary Material

Supplementary material
